# Significance of an increase in the Child-Pugh score after radiotherapy in patients with unresectable hepatocellular carcinoma

**DOI:** 10.1186/1748-717X-9-101

**Published:** 2014-04-29

**Authors:** Seok Hyun Son, Hong Seok Jang, In-Young Jo, Byung Ock Choi, Jeong Won Jang, Seung Kew Yoon, Chul Seung Kay

**Affiliations:** 1 Department of Radiation Oncology, Incheon St. Mary’s Hospital, College of Medicine, The Catholic University of Korea, Incheon, Korea; 2 Department of Radiation Oncology, Seoul St. Mary’s Hospital, College of Medicine, The Catholic University of Korea, Seoul, Korea; 3 Department of Internal Medicine, Incheon St. Mary’s Hospital, College of Medicine, The Catholic University of Korea, Incheon, Korea; 4 Department of Internal Medicine, Seoul St. Mary’s Hospital, College of Medicine, The Catholic University of Korea, Seoul, Korea

**Keywords:** An increase in Child-Pugh score, Radiotherapy, Unresectable hepatocellular carcinoma

## Abstract

**Background:**

We attempted to analyze the effects of an increase in the Child-Pugh (CP) score on the overall survival of patients with unresectable hepatocellular carcinoma (HCC) after radiotherapy (RT).

**Methods:**

From March 2006 to February 2012, 103 patients received RT using the TomoTherapy Hi-Art at Incheon St. Mary’s Hospital and Seoul St. Mary’s Hospital. The dose per fraction was 1.8–5 Gy, and the total dose was 40–60 Gy (median, 50 Gy). We considered an increase of at least 2 points in the CP score within 3 months after RT to be clinically important radiation-induced hepatic toxicity and analyzed the effects of an increased CP score on overall survival.

**Results:**

The median follow-up duration was 11.6 months (range, 3.5–85.3 months). The median survival time was 11.6 months. In multivariate analysis, planning target volume and an increase in the CP score after RT were found to be a statistically significant factors (*p* = 0.010 and 0.015, respectively). In a comparison of cases with and without an increase in the CP score, there was an 11.0-month difference in the median survival time (6.9 vs. 17.9 months), and the relative risk of mortality was 1.8.

**Conclusion:**

An increase of at least 2 points in the CP score within 3 months of RT completion is an important on-treatment factor that affects overall survival. To minimize such increases, careful patient selection and a more sophisticated radiation treatment plan are imperative.

## Background

Radiotherapy (RT) for unresectable hepatocellular carcinoma (HCC) has been used in combination with other local treatments such as transarterial chemoembolization (TACE), percutaneous ethanol injection (PEI), and radiofrequency ablation (RFA) [1-3]. Previously, RT was not widely used because the whole liver could only tolerate low levels of radiation, and these low doses were insufficient to effectively control tumors [4,5]. However, recent studies have shown that partial volume irradiation is both feasible and effective for tumor control with an acceptable range of hepatic toxicity [6-8].

Radiation-induced hepatic toxicity (RIHT) in HCC patients must be considered during RT. Currently, there is no effective treatment for RIHT, which can induce liver failure when not appropriately controlled. Thus, RIHT is considered an important dose-limiting toxicity in HCC patients who receive RT [9]. Therefore, studies have attempted to identify predictive parameters to help reduce the incidence of RIHT, and the results have been helpful in the establishment of radiation treatment plans intended to reduce hepatic toxicity during RT [10-19].

The Child-Pugh (CP) score, which is calculated according to the serum albumin and bilirubin levels, the prothrombin time (PT), and the presence and degree of encephalopathy or ascites, is a system for assessing hepatic function. Therefore, an increase in the CP score might reflect deterioration in hepatic function. Additionally, an increase in the CP score after RT can cause difficulty in administering additional treatments, disease progression or liver failure, which can affect the patient’s prognosis, can be developed [19-21].

In this study, we considered an increase of at least 2 points in the CP score within 3 months after the completion of RT to be an important RIHT. We attempted to analyze the effects of such CP score increases on the overall survival after RT and to discuss its importance.

## Methods

### Patients

The inclusion criteria for this study were as follows: 1) unresectable HCC; 2) age >18 years; 3) a CP score of 5, 6, or 7 within 1 month before RT; 4) an Eastern Cooperative Oncology Group (ECOG) performance status of 0 or 1; 5) an absence of distant metastases; 6) 2 or more laboratory studies within 3 months after the completion of RT; 6) 1 or more radiological studies within 3 months after the completion of RT; and 7) no disease progression within 3 months after the completion of RT.

A total of 103 patients were found to be eligible for this study. All patients received RT using the TomoTherapy Hi-Art system (TomoTherapy Inc., Madison, WI, USA) at Incheon St. Mary’s Hospital and Seoul St. Mary’s Hospital from March 2006 to February 2012. The patient data were retrospectively reviewed following institutional review board approval (IRB of Incheon St. Mary’s Hospital, the Catholic University of Korea, Reference number: OC12RISI0135).

Age, gender, ECOG performance status, American Joint Committee on Cancer (AJCC) stage (7^th^ edition), pretreatment CP score, the absence or presence of hepatitis, liver cirrhosis, or portal vein tumor thrombosis (PVTT), the alpha-fetoprotein (AFP) level, and CP score within 3 months after the completion of RT were evaluated. Before RT, TACE was performed in 95 patients (median number of procedures, 2; range, 1–11), PEI in 8 patients (median number of procedures, 2; range, 1–3), RFA in 8 patients (median number of procedures, 2; range, 1–3), and systemic chemotherapy in 14 patients. The patients’ characteristics are shown in Table 1.

**Table 1:** Clinical characteristics

**Variables**	**n**	**(%)**
Gender		
Male	80	77.7
Female	23	22.3
Age (year)		
Median	59	
Range	21-80	
ECOG		
0	38	36.9
1	65	63.1
Hepatitis		
None	2	1.9
HBV	73	70.9
HCV	9	8.7
NBNC	9	8.7
Alcoholic	10	9.7
Liver cirrhosis		
No	32	31.1
Yes	71	68.9
PVTT		
No	45	43.7
Yes	58	56.3
AFP (IU/mL)		
≤ 400	67	65.0
> 400	36	35.0
Child-Pugh class before radiotherapy		
A	91	88.3
B	12	11.7
AJCC stage		
II	14	13.6
III	81	78.6
IVA	8	7.8
Previous treatment		
None	7	6.8
TACE	95	92.2
RFA	8	7.8
PEI	8	7.8
Chemotherapy	14	13.6

*Abbreviations:ECOG PS* Eastern Cooperative Oncology Group performance status, *HBV* hepatitis B virus, *HCV* hepatitis C virus, *NBNC* non-B, non-C, *PVTT* portal vein tumor thrombosis, *AFP* alpha-fetoprotein, *AJCC* American Joint Committee on Cancer, *TACE* transcarterial chemoembolization, *RFA* radiofrequency ablation, *PEI* percutaneous ethanol injection.

### Radiotherapy

For the simulations, patients were immobilized using the BodyFix system (Medical Intelligence GmbH, Schwabmunchen, Germany), in which the abdomen was compressed under low pressure with foil. Next, a spiral computed tomography (CT) scans were obtained using an intravenous contrast agent and a 2.5-mm slice thickness on either a SOMATOM (Siemens, Berlin, Germany) or a LightSpeed RT16 (GE, Waukesha, WI, USA) CT scanner.

The gross tumor volume (GTV) was defined as the tumor volume that was enhanced in the arterial phase of the CT scan and diluted in the delayed phase. The planning target volume (PTV) was generated by the addition of 5–15 mm to the GTV in 71 of the 103 patients, which facilitated an asymmetric margin expansion in order to reduce irradiation to the stomach, duodenum, and small intestine. In the remaining 32 patients, 4-dimensional (4D) CT was performed to generate an internal target volume in order to compensate for respiration-induced liver movement; these 4D-CT scanners were installed in March 2009 at Seoul St. Mary’s Hospital and in March 2011 at Incheon St. Mary’s Hospital. The organs at risk, such as the total liver, non-target normal liver (NTNL), stomach, duodenum, intestine, kidney, and spinal cord, were also contoured for evaluation of the irradiated dose. The NTNL volume was the total liver volume minus the PTV.

The GTV was 122.8 ± 153.3 cm^3^, the PTV was 330.5 ± 275.1 cm^3^ and the normal liver volume was 1209.7 ± 426.9 cm^3^. The dose per fraction to the PTV was 1.8–5 Gy, and the total dose was 40–60 Gy (median, 50 Gy). The dose was prescribed to 95% of the PTV. The treatment characteristics are shown in Table 2. The prescribed dose varied according to the pretreatment CP class and the PTV. When the PTV was small and the pretreatment CP class was A, the prescribed dose was higher and 4–5 Gy per fraction was used. When the PTV was large or the pretreatment CP class was B, the prescribed dose was lower and 1.8–2.5 Gy per fraction was used. Additionally, normal tissue constraints, which were used in our institution at the time of the study, were applied (Table 3). We intended to prescribe the PTV dose according to the normal tissue constraints; however, these constraints were not always satisfied in order to achieve adequate target coverage and proper tumor dose. Treatment planning was performed using the built-in software of the TomoTherapy Planning Station, which was used with the TomoTherapy Hi-Art system. We evaluated the dose-volume histogram (DVH) and dose distributions in a slice-by-slice manner. We then approved the treatment plan if tumor coverage was adequate and doses to the surrounding normal tissue were within acceptable levels. Megavoltage cone-beam CT was performed during each treatment session before actual beam delivery. Patients’ set-up and position were corrected using automated image registration, and anatomical accuracy was always evaluated by a radiation oncologist.

**Table 2:** Treatment characteristics

**Variables**	
GTV (cm^3^)	122.8 ± 153.3
PTV (cm^3^)	330.5 ± 275.1
Normal liver volume (cm^3^)	1209.7 ± 426.9
Total dose (Gy)	
Median	50
Range	40-60
BED10 (Gy_10_)	
Median	73.5
Range	50.5-82.5
EQD2 (Gy, α/β ratio = 10)	
Median	61.3
Range	42.1-68.8
Hypofractionation (n,%)	
1.8-2.5 Gy per fraction	41 (39.8%)
4–5 Gy per fraction	62 (60.2%)

*Abbreviations*: *GTV* gross tumor volume, *PTV* planning target volume, *BED* biologically effective dose, *EQD2* equivalent dose in 2-Gy fractions.

**Table 3:** Normal tissue constraints

	**1.8-2.0 Gy per fraction**	**2.5-3.0 Gy per fraction**	**4.0-5.0 Gy per fraction**
Liver	TL-V_30Gy_ < 60%		TL-V_20Gy_ < 60%
	Mean dose < 31 Gy	Mean dose < 30 Gy	Mean dose < 22 Gy
Kidney	V_18Gy_ < 33%	Mean dose < 16 Gy	Mean dose < 13 Gy
Spinal cord	D_2cc_ < 45 Gy	D_2cc_ < 42 Gy	D_2cc_ < 33 Gy
Intestine	D_2cc_ < 50 Gy	D_2cc_ < 45 Gy	D_2cc_ < 35 Gy

*Abbreviations:TL* total liver, *V*_*30Gy*_ volume of normal tissue that receives more than 30 Gy, *D*_*2cc*_ maximal dose to 2 cc of normal tissue.

### Evaluation and analysis

We considered an increase of at least 2 points in the CP score within 3 months after the completion of RT to be a clinically important RIHT. The CP score, which is calculated according to the serum bilirubin and albumin levels, the PT, and the presence and degree of ascites or encephalopathy, is used as a tool to assess hepatic function; thus, an increase in the CP score reflects deterioration in the hepatic function [19,21].

The effects of clinical factors, including age, gender, ECOG performance status, the presence or absence of liver cirrhosis, hepatitis, PVTT, AFP level, pretreatment CP class, and PTV, on overall survival were analyzed. The effects of treatment factors, including the biologically effective dose (BED) and fraction size, on overall survival were also analyzed. Furthermore, the effect of an increase of at least 2 points in the CP score within 3 months after RT completion on overall survival after RT was analyzed.

### Statistical analyses

Overall survival was calculated from the date of RT to the date of death or the last follow-up. The probability of cumulative survival was calculated according to the Kaplan-Meier method. Univariate and multivariate analyses were performed according to the Cox proportional hazards models. Multivariate analysis was performed according to the “enter” method. Significant variables in univariate analysis were included in multivariate analysis. The association between the clinical/tumor characteristics and an increase in the CP score was analyzed using the chi-square test and independent t-test. The statistical analysis were performed using STATA 12.1 software (StataCorp, College Station, TX, USA), and *p* values of <0.05 were considered statistically significant.

## Results

### Overall survival and clinical factors that influence the survival

The median follow-up duration was 11.6 months (range, 3.5–85.3 months). The median survival time was 11.6 months, and the 1-, 2-, and 3-year survival rates were 48.5%, 23.4%, and 14.3%, respectively. In univariate analysis, pretreatment CP class B, PTV more than 225 cm^3^, and an increase of at least 2 points in CP score after RT were found to be statistically significant unfavorable factors for overall survival (*p* = 0.038, 0.001, and *p* <0.001, respectively). Gender, age, ECOG performance status, AJCC stage, the level of AFP, the presence or absence of liver cirrhosis, hepatitis, PVTT, BED, and hypofractionation were not found to be statistically significant factors. In multivariate analysis, PTV more than 225 cm^3^ and an increase of at least 2 points in the CP score after RT were found to be statistically significant factors for poor overall survival (*p* = 0.010 and 0.015, respectively). The results of univariate and multivariate analyses are summarized in Table 4.

**Table 4:** Predictive factors that influence the overall mortality

**Variables**	**Univariate analysis**		**Multivariate analysis**	
	**HR (95% CI)**	***p *** **value**	**HR (95% CI)**	***p *** **value**
Gender (female)	1.329	0.262		
	(0.807-2.192)			
Age	1.002	0.826		
	(0.982-1.023)			
ECOG PS (1)	1.052	0.816		
	(0.686-1.613)			
Hepatitis (B)	1.068	0.780		
	(0.672-1.697)			
Liver cirrhosis (presence)	1.371	0.175		
	(0.868-2.164)			
PVTT (presence)	1.457	0.079		
	(0.957-2.217)			
AFP (>400)	1.798	0.083		
	(0.926-3.484)			
Pretreatment CP class (B)	1.834	0.038	1.524	0.255
	(1.033-3.257)		(0.738-3.144)	
PTV (>225 cm^3^)	2.057	0.001	1.821	0.010
	(1.355-3.125)		(1.157-2.865)	
BED (>70 Gy)	0.839	0.448		
	(0.532-1.322)			
Hypofractionation (≧4 Gy per fraction)	0.731	0.146		
	(0.479-1.116)			
An increase in CP score after RT (≥2)	2.283	<0.001	1.795	0.015
	(1.494-3.484)		(1.121-2.881)	

*Abbreviations:HR* hazard ratio, *CI* confidence interval, *ECOG PS* Eastern Cooperative Oncology Group performance status, *PVTT* portal vein tumor thrombosis, *AFP* alpha-fetoprotein, *CP* Child-Pugh, *PTV* planning target volume, *BED* biologically effective dose.

The associations between the clinical/tumor characteristics and an increase of at least 2 points in the CP score are summarized in Table 5. PVTT (*p* = 0.027) and PTV (*p* <0.001) were significantly associated with an increase in the CP score. BED (*p* = 0.066) and hypofractionation (*p* = 0.054) were marginally significant factors that influenced an increase in the CP score.

**Table 5:** Clinical and tumor characteristics assosiated with an increase in CP score

**Variables**	**No toxicity**	**Toxicity**	***p *** **value**
Gender			
Male	45	35	0.475
Female	11	12	
Age			
Median	60	57	0.142
Range	21-80	40-80	
ECOG PS			
0	18	20	0.275
1	38	27	
Hepatitis			
B	40	33	0.892
Others	16	14	
Liver cirrhosis			
No	19	13	0.493
Yes	37	34	
PVTT			
No	30	15	0.027
Yes	26	32	
AFP			
≤400	40	27	0.138
>400	16	20	
Pretreatment CP class			
A	52	39	0.120
B	4	8	
PTV			
≤225 cm^3^	42	18	<0.001
>225 cm^3^	14	29	
BED			
≤70 Gy	22	27	0.066
>70 Gy	34	20	
Hypofractionation			
<4 Gy per fraction	39	24	0.054
≥4 Gy per fraction	17	23	

*Abbreviations:HR* hazard ratio, *CI* confidence interval, *ECOG PS* Eastern Cooperative Oncology Group performance status, *PVTT* portal vein tumor thrombosis, *AFP* alpha-fetoprotein, *CP* Child-Pugh, *PTV* planning target volume, *BED* biologically effective dose.

### Significance of an increase at least 2 points in CP score after the completion of radiotherapy

**Figure 1:**
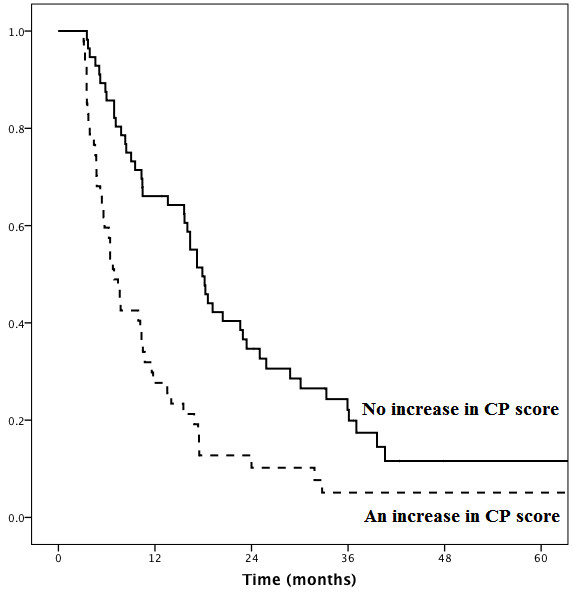
Survival curves according to the presence or absence of an increase in the CP score.

An increase of at least 2 points in the CP score was noted in 47 of the 103 patients (45.6%) after a median time of 1.7 months (range, 0.8–3.0 months) after RT completion. Among these 47 patients, 5 patients (10.6%) recovered from a CP score increase after a median time of 2.73 months (range, 2.53–3.0 months) after RT completion. However, such recoveries were transient because the CP scores increased again after a median time of 0.9 months (range, 0.13–1.13 months). In the absence of an increase of at least 2 points in the CP score, the median survival time was 17.9 months, and the 1-, 2-, and 3-year survival rates were 66.1%, 34.7%, and 22.1%, respectively. In the presence of an increase of at least 2 points in the CP score, the median survival time was 6.9 months, and the 1-, 2-, and 3-year survival rates were 27.7%, 10.2%, and 5.1%, respectively (Figure 1). In a comparison between cases with increased CP scores and those without increased CP scores, there was an 11.0-month difference in the median survival time (6.9 vs. 17.9 months), and the relative risk of mortality was 1.8.

An increase of at least 2 points in the CP score was significantly associated with the number of TACE procedures after RT. TACE could be performed after RT in 64 of 103 patients (62.1%). In cases with no increase in the CP score, the number of TACE procedures was 2.2 ± 2.8; however, in cases with an increase in the CP score, the number of TACE procedures was 1.1 ± 1.1, and this difference was statistically significant (*p* = 0.01). When the analysis was limited to patients who underwent TACE, the number of TACE procedures in patients without an increased CP score (3.7 ± 2.7) was significantly higher than that in patients with an increased CP score (1.7 ± 0.9); this difference was statistically significant (*p* < 0.001). RFA and PEI were performed in 3 and 2 patients, respectively, after RT, and none of these patients experienced an increase in the CP score.

## Discussion

RIHT is an important dose-limiting toxicity in HCC patients that must be considered during RT. Studies have sought predictive factors such as dose-volumetric parameters based on DVH, and these parameters and their values are being used to establish radiation treatment plans. However, the definition of RIHT has varied in previous studies. Radiation-induced liver disease (RILD) is a traditionally accepted concept of hepatic toxicity. Classic RILD is a subacute hepatic toxicity that presents with anicteric ascites, hepatomegaly, and elevated alkaline phosphatase levels; it typically occurs between 4 and 8 weeks after the completion of RT [5,22]. Previously, classic RILD was a serious problem that could occur in response to radiation amounts of 30–35 Gy to the whole liver; however, the incidence decreased after partial volume irradiation became more frequently used [13,14]. Kim *et al.*[10] considered an increase in hepatic enzymes above grade 2 to be RIHT according to the Common Terminology Criteria for Adverse Events (CTCAE) criteria. Dawson *et al.*[18] considered an increase in hepatic enzymes above grade 3 to be RIHT, according to the Radiation Therapy Oncology Group toxicity criteria. Liang *et al.*[12], Lee *et al.*[15], and Cheng *et al.*[16] considered hepatic enzyme levels higher than CTCAE grade 3 to be RIHT and accordingly suggested predictive parameters and their values.

The increase in hepatic enzyme level is temporary, and thus, the levels recover within a few months [10]. In our previous study, the increase in hepatic enzymes could not be appropriately viewed as a dose-limiting toxicity [14]. According to Furuse *et al.*[20], hypoalbuminemia, hyperbilirubinemia, and ascites are important hepatic events that can occur after RT for HCC treatment, and these events can considerably affect patient survival. Albumin, bilirubin, and ascites are the factors used to calculate the CP score. Furthermore, an earlier study stated that progression of the CP class is a useful dose-limiting factor with which to predict the deterioration of hepatic function [14]. An increase of at least 2 points in the CP score was used to evaluate hepatic function deterioration in patients who were treated with lamivudine by Liaw *et al*. [21], and to find a predictive parameter in patients who were treated with helical tomotherapy by Son *et al.*[19]*.* For this reason, the CP score is appropriate for evaluation of hepatic function, and an increase of at least 2 points in the CP score can be viewed as an important factor with which to evaluate hepatic toxicity after RT. Moreover, classic RILD and RIHT-related studies report the occurrence of this toxicity within 3–4 months [10,11,13,17,22]. Therefore, it is reasonable to consider an increase of at least 2 points in the CP score within 3 months to be RIHT.

However, an increased CP score could occur in response to natural deterioration of the underlying liver cirrhosis, tumor progression, and HCC treatment. To minimize the effects of these events on the CP score, a pretreatment CP score of 5–7, an ECOG performance status of 0–1 and the absence of disease progression within 3 months were used as inclusion criteria. Furthermore, the CP score was monitored for 3 months. Although a shorter follow-up duration for the CP score after the completion of RT could reduce the risk of influence of natural deterioration of liver cirrhosis, a 3-month follow-up duration was necessary in order to evaluate RT-induced CP score increases according to previous studies [10,11,13,17,19]. Moreover, we evaluated the laboratory results immediately prior to other treatments to prevent errors caused by the transient elevation of CP scores if additional treatments were performed within the 3-month period after RT.

In our study, PTV of 225 cm^3^ and an increased CP score were found to be significant factors that affected overall survival in multivariate analysis. The pretreatment CP class was a significant factor in univariate analysis, but not in multivariate analysis (*p* = 0.353). According to Cheng *et al.*[16], the risk of RIHT was high in cases of CP class B, and Liang *et al.*[12] reported that the incidence of RIHT was higher in cases of CP class B than in cases of CP class A. However, in a study by Yoon *et al.*[23], the pretreatment CP class was determined to be a factor that affects prognosis in univariate analysis, but not in multivariate analysis. Larger tumor size is a well-known poor prognostic factor in terms of tumor responsiveness or overall survival [6,23,24]. In our study, an increase of at least 2 points in the CP score after the completion of RT was found to be a significant factor instead of the pretreatment CP class. In a comparison of cases with and without an increase in CP score, there was an 11.0-month difference in the median survival time (6.9 vs. 17.9 months), and the relative risk of mortality was 1.8. In a study of Liang *et al.*[13], there was an 18-month difference in the median survival time, which depend on the presence of RILD (4 vs. 22 months). Thus, in that study, the difference in the median survival time was larger than the difference in our study, and the median survival time for the group with hepatic toxicity was shorter (4 months in Liang *et al.* vs. 6.9 months in this study). This might be because Liang *et al.*’s definition of hepatic toxicity was different from ours. Nonetheless, our results are similar in the sense that they show the importance of preventing such toxicity, because the overall survival is low in patients with hepatic toxicity.

Moreover, in this study, the number of TACE procedures performed after RT differed according to whether there was an increase in the CP score (3.7 ± 2.7 vs. 1.7 ± 0.9 times). This could be interpreted to mean that there were fewer opportunities for additional treatments because of hepatic function deterioration in the group with increased CP scores. Unresectable HCC is difficult to treat completely with RT alone; therefore, treatments such as TACE are repeatedly performed to obtain the maximum effect. However, if it becomes difficult to administer such procedures because of hepatic function deterioration, patient survival may be adversely affected because there are few other opportunities to control tumor progression.

In conclusion, an increase of at least 2 points in the CP score within 3 months of RT completion is an important on-treatment factor that affects overall survival. To minimize such increases, careful patient selection and a more sophisticated radiation treatment plan are imperative.

## Competing interests

The authors declare that they have no competing interests.

## Authors’ contributions

SHS, CSK, IJ, BOC, and HSJ collected the clinical data and interpreted the results. SHS, CSK, HSJ, JWJ, and SKY cared for the patients. SHS, CSK, JWJ, SKY, and HSJ were involved in the study design. SHS performed the statistical analysis and drafted the manuscript. All of the authors have read and approved the final draft.
